# Supplementary material for: Task-anchored grid cell firing is selectively associated with successful path integration-dependent behaviour
Source: eLife. 2024 Mar 28;12:RP89356. doi: 10.7554/eLife.89356 (PMC10977970; doi:10.7554/eLife.89356)
Supplement: Supplementary file 1. [file elife-89356-supp1.docx]

| Mouse | N cells | N sessions | N trials / session | N grid cells (%) | Tetrode location |
| --- | --- | --- | --- | --- | --- |
| M1 | 52 | 11 | 45.5 ± 33.6 | 3 (5.8) | MEC |
| M3 | 243 | 26 | 270.3 ± 93.5 | 7 (2.9) | MEC |
| M6 | 229 | 21 | 172.3 ± 99.8 | 1 (0.4) | MEC |
| M7 | 191 | 23 | 268.1 ± 137.8 | 4 (2.1) | MEC |
| M10 | 66 | 16 | 74.3 ± 34.8 | 1 (1.5) | Unclassified |
| M11 | 557 | 35 | 173.1 ± 126.1 | 53 (9.5) | MEC |
| M12 | 105 | 21 | 244.4 ± 68.5 | 1 (0.95) | Unclassified |
| M13 | 160 | 25 | 47.5 ± 25.1 | 7 (4.3) | MEC |
| M14 | 278 | 37 | 316.4 ± 148.2 | 26 (9.4) | MEC |
| Average | 209 ± 152.5 | 23.9 ± 8.3 | 179.1 ± 103.4 | 11.4 ± 17.4  (4.5 ± 3.5) |  |
| Total | 1881 | 215 |  | 103 |  |

**Summary table of recorded cells and estimated tetrode locations.** Tetrode locations were estimated from microCT images as Supplemental Data 2. For average measurements, standard deviations are provided. Mice used in Tennant et al. 2022 were M3, M6 and M7.
