# Supplementary figures and images for: Task-anchored grid cell firing is selectively associated with successful path integration-dependent behaviour
Source: eLife. 2024 Mar 28;12:RP89356. doi: 10.7554/eLife.89356 (PMC10977970; doi:10.7554/eLife.89356)

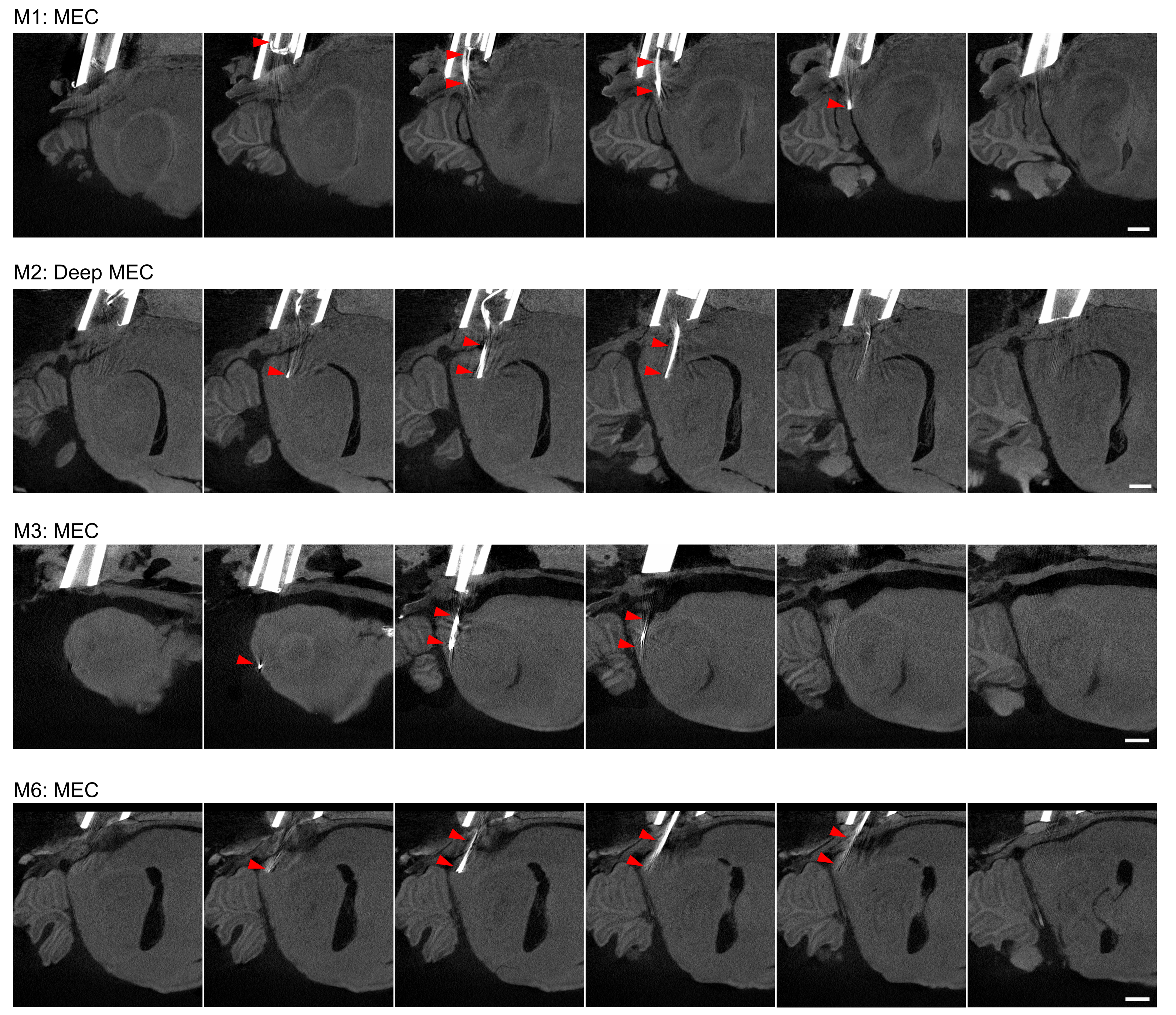

Supplement: Source data 1. [file elife-89356-data1.png]

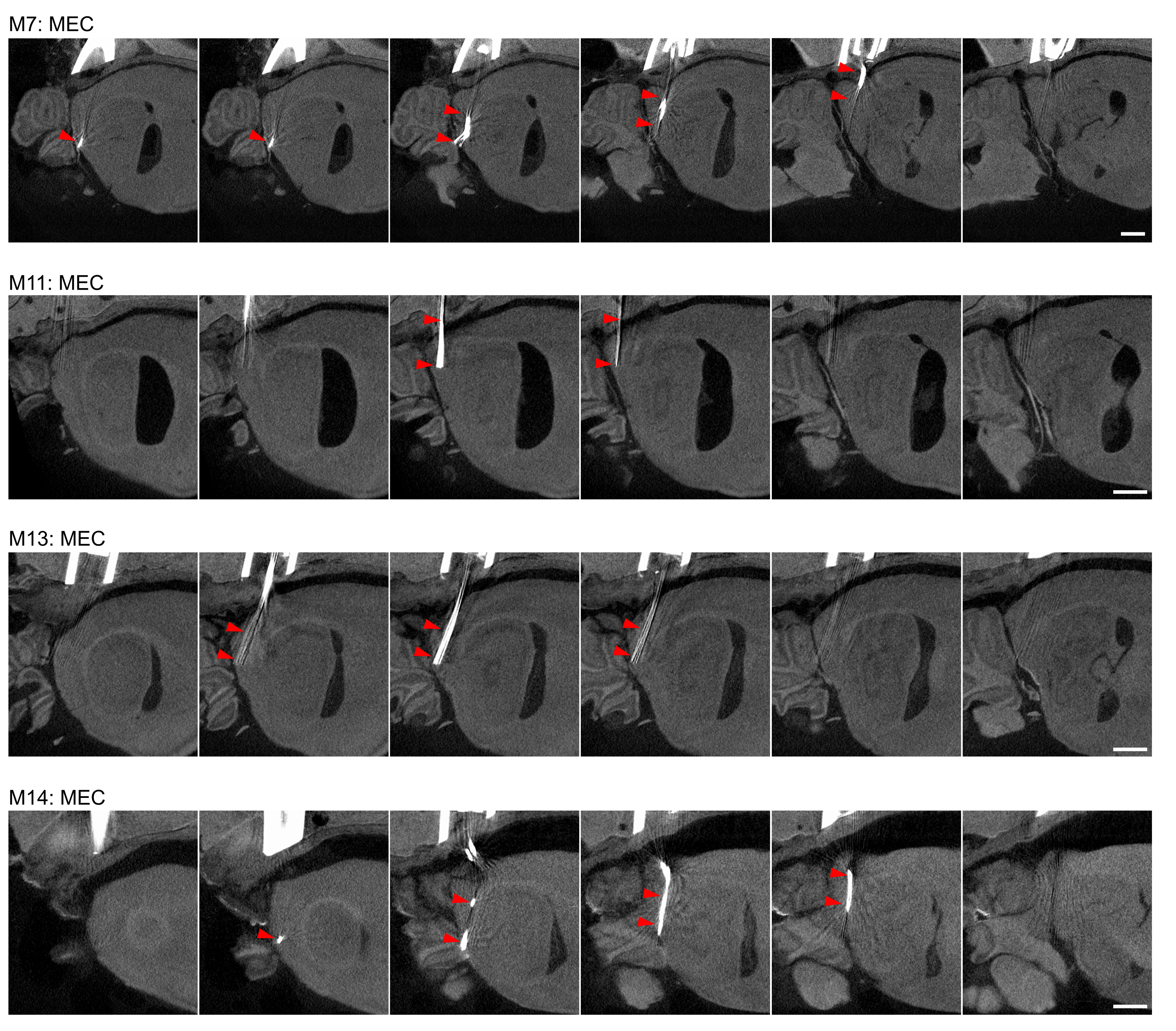

Supplement: Source data 2. [file elife-89356-data2.png]
